# Quality of Care in Family Planning Services: Differences Between Formal and Informal Settlements of Kira Municipality, Uganda

**DOI:** 10.3389/fgwh.2021.656616

**Published:** 2021-08-13

**Authors:** Zubair Lukyamuzi, Moses Tetui, Osvaldo Fonseca-Rodríguez, Lynn Atuyambe, Fredrick Edward Makumbi, Mazen Baroudi

**Affiliations:** ^1^Makerere University, Johns Hopkins University Collaboration (MU-JHU), Kampala, Uganda; ^2^School of Pharmacy, Waterloo University, Waterloo, ON, Canada; ^3^Department of Epidemiology and Global Health, Umeå University, Umeå, Sweden; ^4^Department of Health Policy, Planning and Management, Makerere University School of Public Health, Kampala, Uganda; ^5^Centre for Demographic and Ageing Research, Umeå University, Umeå, Sweden; ^6^Department of Community Health and Behavioural Sciences Makerere University School of Public Health, Kampala, Uganda; ^7^Department of Epidemiology and Biostatistics, Makerere University School of Public Health, Kampala, Uganda

**Keywords:** family planning services, quality of health care, urban health, informal settlements, Uganda

## Abstract

**Background:** Quality of care (QoC) of family planning (FP) affects contraceptive use, and it varies across types of urban settlement. This study assesses the difference in service delivery point (SDP) structural and process factors between formal and informal urban settlements, and the opinion of the client on the QoC in informal settlements. This is useful in creating an evidence base to advocate for better quality services for the most vulnerable in society.

**Methods:** This was a cross-sectional survey that included SDPs of Kira municipality in Wakiso district, Uganda. Data were collected from all the service points in Kira municipality with the caretakers consented. In addition, using multi-stage sampling, 626 women of reproductive age (15–49 years) who lived in the informal settlements of Kira municipality were interviewed. Data were collected using structured questionnaires, descriptive analysis was carried out in Stata version 14.0, and Chi-square and *t*-tests were used to compare the informal with the formal settlements.

**Results:** Formal settlements generally had more higher-level SDPs compared to informal settlements (value of *p* < 0.001). SDPs in the formal settlements provided more FP methods and had more community health workers (CHW) to support their work. Also, SDPs in the formal settlements were more likely to have long-term FP methods available and more likely to have trained personnel to insert and remove implants and IUDs compared to those in informal settlements. Additionally, more SDPs in the formal settlements provided counseling for permanent, long-term, and short-term FP methods. Of the 626 interviewed women, most of the women (68.6%) reported that they would not return to the previous FP provider or refer a friend to the same provider (72.7%).

**Conclusions:** There is a lower quality FP services in the informal settlements with a commensurable effect on the client satisfaction with the services. Therefore, improving the quality of FP services in informal settlements should be a top priority. Improved quality of services could act as a motivation to increase the uptake of modern contraceptives in such settings.

## Introduction

In comparison to other regions, decline in fertility levels has been slower in sub-Saharan Africa (SSA) (1). The fertility rate in SSA was estimated to 5.1 births per woman in the period, 2010–2015 and projected to be 4.75 in the period, 2015–2020 (2), whereas the fertility rates are close to the desired 2.1 children per woman in Asia, Latin America, and the Caribbean for the same periods (3–5). The high fertility rate is associated with increased maternal mortality, neonatal mortality, and under-five mortality (6, 7). One of the major strategies for fertility reduction is the use of family planning (FP) methods, particularly modern contraception (8); however, over 225 million women are estimated to have an unmet need for FP, more so in sub-Saharan Africa (9, 10).

In Uganda, however the percentage of women aged 15–49 who use a modern contraceptive method has increased over the years, but still remains relatively low at 35% as of 2016 (11). The total fertility rate of the country remains one of the highest worldwide at 5.4 children per woman, with 28% of currently married women and 32% of sexually active unmarried women having an unmet need for FP. Moreover, 45% of contraceptive discontinuations were estimated by the 2016 Demographic and Health Survey (DHS) (11). Unmet need for contraception and contraceptive discontinuation can be due to several obstacles including, but not limited to, lack of knowledge of contraceptive methods, concerns of side effects, stock-outs, as well as low quality of FP services (9, 11).

High quality of care (QoC) in FP services is associated with increased and continued contraceptive use in several settings (12–16). A multi-country analysis based on DHS data in 15 countries showed that about 7–27% of women discontinue contraception because of a reason related to the QoC (12). In a longitudinal analysis in Bangladesh, Koenig et al. (16) found that women had a 60% higher likelihood of adopting a modern method and a one-third lower likelihood of discontinuing the method if they received a higher quality of FP services from fieldworkers. Similarly in the Philippines, the quality of FP services women received determined maintenance and continuation of the method (17). Research in Uganda has also found that the quality of FP services affects significantly their utilization by clients (18). More specifically, providing counseling to the clients during FP services was found to improve both long-term outcomes, such as increased birth spacing and continued use of modern contraception methods, as well as short-term outcomes such as increased knowledge and satisfaction with the FP services (19–21).

Despite the importance of quality in providing FP services, measuring the QoC can be challenging due to various definitions and components. Donabedian (22) discusses the measurement of QoC in three components, namely, structure (including the infrastructure of the health facility, equipment, and its commodities), process (the health provider's method of delivering care including interpersonal interaction with the client and technical competency), and outcome (the outcome of the service provided such as satisfaction of the clients). Based on the framework of Donabedian, recent literature in SSA and planning monitoring and accountability (PMA 2020) has synthesized three pillars of FP QoC as measured through the client satisfaction, and these are (1) socio-demographic characteristics of clients, providers, and facilities, (2) structural factors such as staffing, and (3) process factors such as provider–client interaction (13, 22–29) as shown in Table 1.

**Table 1:** A modified framework of quality of family planning services according to PMA 2020.

	**Examples**	**Relevant PMA 2020 variables**
Socio-demographic factors	Age, gender, and education status of the client Provider's experience and FP training Type, level, ownership, and location of the health facility	- Age of the client - Provider's experience and knowledge in FP - Facility ownership - Facility level
Structural factors	Facility human resources, supervision, distance, business hours, cleanliness, infrastructure, availability of contraceptive methods and equipments, and fees	- Number of staff - SDP supports CHWs - Number of days of offering FP - Examining room cleanliness - Electricity, water, and handwashing availability in the facility - Fees charged for FP - Methods of FP provided - Methods of FP out of stock - Examining room supplies
Process factors	Client provider interaction (communication, choice of method, information provided), confidentiality, client waiting time, pre-requite for contraceptive services	- Visual and auditory privacy - Collection and review of client opinions - Provision of services to adolescents - Provision of post -abortion services

*FP, family planning; SDP, service delivery point; CHWs, community health workers*.

The information required to understand the above aspects of QoC in FP can be obtained by either interviewing the clients using the services or through observing the interactions between the provider and the client. However, a combination of observations and client interviews is suggested to provide a better assessment (30).

Quality of care is usually perceived to be better in urban compared to rural settings. However, with increasing urbanization, it is important to examine the QoC in such settings, given that 68% of the population of the world will be living in urban areas by 2050 (31). Indeed, there is an empirical generalization that the fertility levels tend to be positively related to urbanization since most fertility indicators are better in urban areas than in rural areas. However, this gap is narrowing in many countries due, partly, to significantly worse outcomes among the urban poor living in the informal settlements (32). As opposed to the formal settlements, the informal settlements commonly known as slums are characterized by inadequate housing, insufficient living spaces, insecure land tenure, and lack of access to basic services including social services such as clean water and sanitation (33). Urban women in the highest wealth quintile, the use of contraception is two and half times more compared to urban women in the poorest wealth quintile; and a similar pattern emerges with respect to the unmet need for FP (32). This study sets out to assess the QoC of FP services at a service delivery point (SDP) with a focus on provider-related structural and process factors exploring variations between the informal and the formal settlements of Kira municipality. The study also assesses the FP service satisfaction among the women living in the informal settlements.

## Materials and Methods

### Study Design and Settings

This was a cross-sectional survey for all health facilities in Kira municipality. Kira is located in Wakiso District of the Central Region of Uganda and is the second most populated municipality in the country. Kira municipality is made up of three divisions, namely, Kira, Bweyogerere, and Namugongo, which occupy a total land area of about 98.83 km^2^. The rapid population growth in this area has in-turn compromised physical planning and effective service delivery including healthcare. This has eventually resulted in the growth of the informal settlements, whose inhabitants have a low socio-economic status and reside in poor household structures. The municipality headquarters are located about 10 km from the city center with a growing population of 400,000 people, of whom 47.8% are men and 52.2% are women (34, 35).

### Description of Family Planning Services in Kira Municipality

Family planning services in Kira municipality are provided by various health facilities, including drug shops, health centers, and pharmacies. Some facilities are supported by the Ministry of Health (MOH) or non-government organizations (NGOs), and others are privately owned. FP is provided on a specific day and specific time in some facilities while other facilities do not have a specified day or specified time. FP services provided in the facilities range from short-term methods to permanent methods (36).

### Study Population and Sampling Procedures

To measure the structural and process factors of quality of care, we surveyed all family planning service points at different levels of service in the Kira municipality, and these included general hospitals, health centers at level IV (managed by a doctor, and surgeries are carried out), level III (managed by a clinical officer and offers inpatient care), and level II (managed by a nurse and offers only outpatient care) (37), pharmacies, clinics, and drug shops and their caretakers. Health facilities whose caretakers consented (187 out of 192 facilities) were mapped and targeted for the survey.

In addition, women of reproductive age (14–48) were interviewed to assess the perception of the client about the FP services in the informal settlements. A total of 626 women who lived in the informal settlements of the study area for at least 6 months were randomly sampled and recruited in the study. Multi-stage sampling was conducted. First, four out of the eight villages of the informal settlements of Kira municipality were randomly selected. Thereafter, 13 of the 65 Enumeration Areas (EAs) within these villages were randomly selected. Finally, the participants were randomly selected from a list of all households that had eligible women. The sample size for women respondents in this study was calculated primarily to measure the prevalence of contraceptive use and unmet need for family planning in this setting (38) using the Kish Leslie formula for cross-sectional studies, *N* = $\frac{Z^{2}PQ}{\sigma^{2}}$. We assumed a prevalence of modern contraceptive use in an urban setting in Uganda of 52.1% (11), a 95% level of confidence, a margin of error (d) of 0.05, and a Z score of 1.96 after adjusting for no response and design effect. The total size of the sample was 626 participants and the potential participants were selected using the multi-stage sampling through their enumeration areas and households.

### Data Collection

Data for this analysis are a part of an earlier survey conducted to determine the unmet need for FP in informal settings (38). The data were collected by trained research assistants, who were familiar with Kira municipality. In addition, local guides who worked at the municipality supported the research assistants to easily identify the facilities for the mapping exercise and potential women for study participation. KoboCollect ( support@kobotoolbox.org), a mobile data collection application was used to collect the data. Before the data collection, the questionnaires (assessing the structural and process factors affecting the quality of FP at the SDP as well as the opinion of the women on the FP delivery) were initially pre-tested around the Makerere University area; the area has both the formal and the informal settlements, which made it ideal, given its similarities with the actual study area. Following the pre-test, to ensure quality quantitative data collection, the data entry screen was designed with skips and more restrictions added to ensure completeness of entry. On a daily basis, the completed questionnaires were uploaded to a remote server to which the PI and the study coordinator had access. The data were kept strictly confidential and later analyzed after de-identification.

### Measures

For this analysis, the main outcome was the quality of FP services in Kira municipality, by comparing the informal and the formal settlement. A secondary outcome was the opinion of the user (women) about the quality of FP services from their respective SDPs. Quality of FP services was determined as the availability of provider-related structural and process measures at a given facility with respect to client satisfaction.

### Statistical Analysis

Data analysis was performed with Stata version 14.0 (Stata Corp) software. Descriptive statistics for the baseline characteristics were conducted and the analysis was presented with summaries in percentages for the categorical variables, and mean (±SD) for continuous data. A comparison of key outcomes for the informal and the formal settlements was conducted using the Chi-square for categorical variables, and the *t*-test for the continuous variables, with a *p*-value at <5% for statistical significance cut-off. The opinion of the women on FP services was also described in proportions.

## Results

### Characteristics of Health Facilities in Kira Municipality

In Kira municipality, the majority of the health facilities in the informal settlements had clinics with one or two rooms (53.8%) and only 21.3% of the clinic had at least three rooms, drug shops (20%), health center III (2.5%), and health center II (1.3%) whereas, in the formal settlements, most of the health facilities had clinics 3+ rooms (29.0%), followed by pharmacies (23.4%), clinics with one to two rooms (16.2%), drug shops (16.2%) and health center III (10.3%).

In general, there were no statistical differences in facility ownership between the informal and formal settlements. However, the difference between the number of private health facilities in the formal settlements and the informal settlements should not be neglected, showing a barely significant *p*-value (*p* = 0.051); we found more private health facilities in the formal settlements. In addition, most of the facilities were privately owned (95.2%) and the majority (94.1%) of the facilities provided the FP services. Moreover, there was a statistically significant higher number of facilities that offered FP services in the formal settlements compared to the informal settlements, as shown in Table 2.

**Table 2:** Characteristics of health facilities in Kira municipality.

	**All facilities**	**Informal settlements**	**Formal settlements**	***p*-value**
	***N* = 187**	***N* = 80**	***N* = 107**	
	**%**	**%**	**%**	
**Health facility level**				
1–2 roomed clinic	32.6	53.8	16.2	0.001
3+ roomed Clinic	25.7	21.3	29	0.043
Drug shop	18.2	20	16.2	0.731
Pharmacy	13.4	0	23.4	N/A
Health center II	1.1	1.3	1	N/A
Health center III	7	2.5	10.3	0.013
Health center IV	1.6	1.3	1.9	N/A
Hospital	0.5	0	1	N/A
**Health facility ownership**				
Faith-based organization	1.6	1.3	1.9	
Government	2.1	2.5	1.9	N/A
NGO	1.1	1.3	1	N/A
Private	95.2	95	93	0.051
**Facility offered FP services**	94.1	92.5	95.3	0.035

*N/A, very few observations to get a p-value*.

### Provision of Family Services at the Health Facilities in Kira Municipality

#### Structural Factors

Overall, the mean number of FP methods provided by facilities was (3.7 ± 1.70), and facilities in the formal settlements provided significantly more FP methods (4.1 ± 1.78) compared to those in the informal settlements (3.3 ± 1.46). Health facilities in the formal settlements were supported by more number of CHWs in providing FP services (3 ± 1.58) compared to the informal settlements (1 ± 0.45), and long-term FP methods were significantly more available in the formal settlement facilities (26.2%) compared to the informal settlement facilities (8.8%). More facilities in the formal settlements had the requirements to insert and remove the implant (30.0%) and IUD (21.5%) compared to the informal settlements (10.8%) and (3.8%), respectively, and showing significant differences in both cases. Similarly, more facilities in the formal settlements had more trained personnel to insert and remove the implant (33.6%) and IUD (25.2%) compared to the informal settlements (14.8%) and (5.0%), respectively (Table 3).

**Table 3:** Family planning services provided at the facilities.

**Characteristics**	**All facilities *N* =1 87**	**Informal settlements *N* = 80**	**Formal settlements *N* =1 07**	***p*-value**
**Structural factors**				
Number FP methods provided by a health facility; mean (±SD)	3.7(±1.70)	3.3(±1.46)	4.1(±1.78)	0.001
Offering FP services for ≥ 6 days a week; (%)	81.3	78.4	83.3	0.406
Providing ≥ 3 methods of FP; (%)	51.1	46.0	54.9	0.241
Using CHWs in providing FP; (%)	5.7	6.8	4.9	0.6
Number of CHWs used per facility for FP; mean (±SD)	2.1(±1.46)	1.2(±0.45)	3(±1.58)	0.02
Availability of long term FP; (%)	18.7	8.8	26.2	0.003
Offering permanent FP methods; (%)	1.1	1.4	1.0	0.819
Having the requirements to insert and remove IUD; (%)	14.0	3.8	21.5	0.001
Having the requirements to insert and remove implant; (%)	21.4	10.8	30.0	0.004
Availability of short term common FP; (%)	48.9	45.0	51.4	0.642
Having trained personnel to insert and remove implant; (%)	25.1	13.8	33.6	0.008
Having trained personnel to insert and remove IUD; (%)	16.6	5.0	25.2	<0.001
Having an EMR where FP is conducted; (%)	48.1	43.8	51.4	0.494
EMR having water and accompaniments; (%)	5.9	6.3	5.6	0.524
EMR having waste disposal equipment; (%)	26.7	26.3	27.1	0.468
EMR having requirements for patient examination; (%)	33.7	35.0	32.7	0.156
**Process factors**				
Providing adolescent FP services; (%)	75.6	74.3	76.5	0.744
Providing FP outreaches; (%)	9.1	6.8	10.8	0.359
Number of FP outreaches by facilities; mean (±SD)	1.2(±0.89)	1(±0)	1.3(±1.12)	0.263
Counseling for permanent FP methods; (%)	18.8	10.8	24.5	0.022
Counseling for long term non-permanent FP methods; (%)	36.9	21.6	48.0	<0.001
Counseling for short term common FP; (%)	48.9	39.2	55.9	0.029
EMR having privacy; (%)	27.3	31.3	24.3	0.05

*FP, family planning; CHW, community health worker; IUD, intrauterine device; EMR, examination room; SD, standard deviation*.

#### Process Factors

Significantly more facilities in the formal settlements provided counseling for permanent FP methods (24.5%) compared to those in the informal settlements (10.8%), and more facilities in the formal settlements offered counseling for long-term FP (48.0%) compared to those in the informal settlements (21.6%). Likewise, more facilities in the formal settlements (55.9%) provided counseling for short-term FP compared to) the informal settlements (39.2%) (Table 3).

#### Client Opinions

A total of 626 women were interviewed in the survey. The mean (SD) age of the participants was 28.1 (±7.6) years. The adolescents and young people aged 15–24 years represented 36.1% of all women in the survey. Most (55.9%) respondents had attended secondary or a higher level of education, and nearly 75% were currently married or living with a man.

The majority of the participants reported that they would not return to the previous FP provider (68.6%) or refer a relative or a friend to the very provider (72.7%). The majority of the participants (83.4%) reported having told about FP when they visited the health facility for other services. Most of the participants (77.3%) reported having been visited by a CHW for FP services, as shown in Table 4.

**Table 4:** The opinion of participants on family planning services in Kira municipality.

**Client opinions**	***N* = 626**
	**(%)**
Returning to the provider	
Yes	7.6
No	68.6
Don't know	10.7
No response	13.1
Refer a friend or relative to the provider	
Yes	7.2
No	72.7
Don't know	13.5
No response	6.7
Told about FP when I visited the facility for other issues	
Yes	83.4
No	16.6
Paid fees for FP at the facility	
Yes	50
No	50
Visited by a CHW for FP	
Yes	77.3
No	22.7

*FP, family planning; CHW, community health worker*.

## Discussion

Overall, most of the surveyed facilities were privately owned, and the majority of them provided FP services. The formal settlements had more health facilities and a higher level of facilities compared to the informal settlements. Facilities in the formal settlements provided more FP methods, used more CHWs were more likely to have long-term FP methods available, and trained personnel to insert and remove implants and IUDs compared to those in the informal settlements. Despite more facilities in the formal settlements providing counseling for permanent, long-term, and short-term FP methods, the difference was smallest for short-term methods. Finally, although most of the participants reporting to have been told about FP when they visited a health facility for other services and the majority were visited by a CHW for FP services, the majority of the participants reported that they would not return to their previous FP provider and would not refer a relative or friend to that provider.

Generally, the quality and availability of FP services were less in the informal settlements compared to the formal settlements. A similar observation was reported in Bangladesh (39, 40). Relatedly in Uganda, sexual and reproductive health were reported to be poorer among the urban poor (41). In this study, levels of health facilities were lower in the slum areas; health facilities in the informal settlement provided less number of methods and had less number of methods available. These results reflect that the urban poor women often live in informal settlements that are largely excluded from the formal services (42). Previous studies have shown that many health facilities in informal settlements are private and usually lack basic resources such as diagnostic equipment, supplies, and medicines. In Bangladesh, for example, most of the dwellers of the informal settlement were reported to get their health services from the informal health facilities such as drug shops (43). Therefore, the dwellers of the informal settlement are reported to rely on such facilities because the public health facilities closest to them are also in poorer conditions. However, in addition to being in a poor condition, private clinics charge high fees for FP services and hence making contraception in informal settlements less affordable and utilized (32, 44). For instance, in Kenya, women living in informal settlements were less likely to use short-term or long-term methods compared to their counterparts living in formal settlements (45).

Despite the weak positive association between the number of staff in a health facility and client satisfaction (25), the number of trained personnel available is critical in defining the quality of FP services (23). In our study, informal settlement facilities had less number of trained personnel to insert and remove the implants and IUDs compared to those in the formal settlements. This is similar to Oketch et al. (46) findings in Kenya in which they reported that informal settlements are likely to be served by informal providers that are likely to be poorly regulated and hence provide poor quality of services. In our study, the lesser number of trained personnel to insert and remove the implants or IUDs in slum areas may be generally explained by the significantly declining provision of health services in the public sector, which is known to use trained health providers (47). The declining provision of health services in the public sector leads to an increase in private providers who can be difficult to regulate especially in emphasizing professionalism in providing the services. In Uganda particularly, 80% of contraceptive users obtained their methods from a public facility in 1988 and by 2006 this proportion had decreased to as low as 34% (32, 47).

Generally, the average proportion of counseling for all FP methods was low at 34.9%. However, this was higher than what was reported in India from a longitudinal household survey for which 22% of interviewed women aged 15–49 reported to have received FP counseling (48). This difference could probably be due to different study designs and populations. The Indian study used a longitudinal survey and included only those women who were currently on modern contraception as opposed to our study which used a cross-section design and inclusion of all women of reproductive age (15–49 years). Even though the provision of FP counseling for all methods (short-term, long-term, and permanent) was less in the informal settlements compared to the formal settlements, the gap was smallest for short-term methods. This implies a significant deficit in the use of long-term and permanent methods in the informal settlements, yet dwellers of such settlements need more reliable and easy compliance methods due to the higher fertility rate associated with the poor communities (32). Similar to our findings, a study conducted in Kenya showed that the women in slum areas choose more short-term contraceptives compared to the long-term methods (45). This can be due to the long-term methods being more expensive than the short-term; hence, it is less affordable by women in the poorest wealth quintile in the informal settlements (49). Low levels of counseling, especially on long-term and permanent FP methods, may lead to persistence in localized social networks of the urban poor, which may serve to sustain and foster misconception about the contraceptive methods and limit information about the availability of FP services hence undermining the use of contraception with an eventual increase of unmet need for contraception.

In this study, more participants reported having been told about FP when they visited the health facility for other services (83.4%) and were visited by a CHW for FP services (77.3%). This implies that in Uganda, women are likely to get significant information about FP, which is reflected in the 2016 DHS finding, reporting that 99% of women aged 15–49 had general knowledge about FP (11).

Although significant FP service client satisfaction was reported in previous intervention research studies in Uganda (50–52), our findings show that this may not be a similar picture in a program setting. This is because most of the participants in our study reported that they would not return to their previous FP provider and would not refer a relative or friend to that FP provider. Mindfully, the perceived quality of services is considered to be a predictor of client satisfaction (53, 54). Moreover, in our study, all the quality defining parameters of quality of care at SDPs were generally below 50% and worse in slum areas; this may imply a commensurable effect on the client satisfaction with the services in the slum areas. The discrepancy in previous interventional research studies (50–52) and our findings (program setting) could be because, in research settings, participants receive better care than clients in a program setting. However, our findings are also discrepant from Akol A et al. findings (55), in rural districts of Uganda (program setting) reported that most of the Depot-medroxyprogesterone acetate (DMPA) clients (74%) were very satisfied receiving their method from the drug shop, and 98% intended to get their next injection from the same type of facility. This higher satisfaction in Akol A et al. (55) study could be due to the fact that the drug shop attendants in this study had been just trained to offer DMPA 1 year before the evaluation survey. Implying that if FP providers are trained, clients can get satisfied with the services.

### Strengths and Limitations

We were not able to adjust for other factors in the analysis due to the low sample size and the many indicators of quality we were using. Controlling for other factors such as the level and the ownership of facilities could potentially change the results. The complexity of defining quality of care, as well as selecting and constructing the indicators, is another limitation of the study. Lastly, satisfaction is always subjective and may not easily be generalized. The strength of the study is the inclusion of all health facilities in the municipality; therefore, we think that our findings may be generalizable to similar town settings.

### Conclusion and Recommendations

Indicators of FP quality of care were less available or provided in the informal settlement-based facilities compared to the formal settlement-based facilities. Clients in the informal settlements were generally not satisfied with the FP services despite significant FP information. Additionally, FP services in the informal settlements have lower quality services and hence need to be improved to fulfill the contraceptive demands of people in such settlements.

Since most of the urban residents in the least developed countries live in the informal settlements, there is a need to reduce existing inequalities in the provision of FP services. Therefore, policies should not be made based on urban averages alone, but instead, priority should be given to the specific needs of the poor and vulnerable people in the informal settlements. This can be achieved by training health care providers in the informal settlements to provide appropriate and adequate counseling to increase client satisfaction. Furthermore, the government should establish a partnership with development partners such as NGOs and private facilities in providing subsidized professional and quality FP services, especially long-term FP to the urban poor living in the informal settlements.

## Data Availability Statement

The raw data supporting the conclusions of this article will be made available by the authors, without undue reservation.

## Ethics Statement

The study involved human participants and it was reviewed and approved by the Ethics Committee of Makerere University School of Public Health higher degrees (HDREC-684). The patients/participants provided their written informed consent to participate in this study.

## Author Contributions

ZL led the the data analysis and drafting of the manuscript. MT led the project, participated in data collection and supported data analysis. OFR supported data management. LA and FEM supported the conceptualization of the project and data collection. MB supported the data analysis and provided overall technical guidance to the conceptualization and drafting of the manuscript. All authors contributed to the article and approved the submitted version.

## Conflict of Interest

The authors declare that the research was conducted in the absence of any commercial or financial relationships that could be construed as a potential conflict of interest.

## Publisher's Note

All claims expressed in this article are solely those of the authors and do not necessarily represent those of their affiliated organizations, or those of the publisher, the editors and the reviewers. Any product that may be evaluated in this article, or claim that may be made by its manufacturer, is not guaranteed or endorsed by the publisher.
